# Distal third diaphyseal humeral fractures treated with posterior minimally invasive plate osteosynthesis (MIPO)

**DOI:** 10.1016/j.tcr.2026.101342

**Published:** 2026-04-25

**Authors:** Edgar Barros Prieto, Carlos Ballesteros Ponce, Carlos Eduardo Noboa Freile, Carlos Peñaherrera Carrillo, Francisco Endara, Paul Santiago Vaca, Diego Michilena, Alejandro Xavier Barros Castro

**Affiliations:** a Department of Orthopedics and Traumatology, Hospital Voz Andes, Quito, Ecuador; b Department of Orthopedics and Traumatology, Hospital Metropolitano, Quito, Ecuador; c Department of Orthopedics and Traumatology, Hospital Axxis, Quito, Ecuador; d Department of Orthopedics and Traumatology, Hospital Voz Andes and Hospital Metropolitano, Quito, Ecuador; e Postgraduate Program in Orthopedics and Traumatology, International University of Ecuador, Hospital Metropolitano, Quito, Ecuador

**Keywords:** Humeral fractures, Minimally invasive surgical procedures, Fracture fixation, Nerve injury, Radial nerve, Plate osteosynthesis, Outcome assessment, Postoperative complications

## Abstract

**Objectives:**

To describe the technique, safety, and clinical outcomes of posterior minimally invasive plate osteosynthesis (MIPO) in patients with distal third diaphyseal humeral fractures, highlighting its advantages over traditional approaches.

**Methods:**

This retrospective case series included four patients with distal third diaphyseal humeral fractures (AO/OTA 1.2.A-c and 1.2.B-c) treated using posterior MIPO. Patients aged 18–70 years, without prior radial nerve injuries, were included. The procedure involved two small longitudinal incisions, nerve protection, and submuscular plate placement with indirect reduction techniques. Functional outcomes were assessed using VAS, DASH, Constant Score, and MEPI at 1 month, 6 months, and 12 months postoperatively. Radiographic alignment and complications were also evaluated.

**Results:**

Four patients (mean age 45 years) were treated. The average surgical time was 1 h 30 min. No infections or cases of nonunion were observed. Only one patient developed transient radial nerve paresthesia, which resolved completely within 4 weeks. Functional outcomes were excellent at final follow-up (12–36 months): **VAS**: 0–2;**DASH**: 0–5; **Constant Score**: 95–100; **MEPI**: 90–100. Radiographs demonstrated fracture union at an average of 4 months, with residual varus angulation between 0° and 5° without functional impact.

**Conclusions:**

Posterior MIPO is a safe and effective technique for distal third diaphyseal humeral fractures, offering excellent functional outcomes, low complication rates, and reduced soft tissue disruption. Proper identification and protection of the radial nerve are critical to success.

## Introduction

Distal third humeral shaft fractures represent a significant clinical and surgical challenge, accounting for approximately 3% of all fractures with an estimated incidence of 25 cases per 100,000 individuals annually [1], [2]. These fractures demonstrate a bimodal distribution: they predominantly affect women over 50 years of age, due to osteoporosis and bone fragility, and young males involved in high-energy trauma [2], [3]. In older populations, low-energy falls are the most common mechanism of injury, highlighting their association with fragility fractures [3], [4].

While conservative treatment with functional bracing remains the first-line option for stable fractures, surgical indications include open fractures, vascular injuries, polytrauma, and failure of conservative management [1], [5]. Among surgical options, Minimally Invasive Plate Osteosynthesis (MIPO) has gained prominence due to its ability to preserve soft tissues and reduce complications. MIPO has demonstrated clear advantages over Open Reduction and Internal Fixation (ORIF) and Intramedullary Nailing (IMN), including lower rates of nonunion, radial nerve palsy, and postoperative complications, while yielding superior functional outcomes [5], [6], [7].

Most existing literature describes anterolateral or lateral approaches for MIPO, leaving the posterior approach underexplored despite its anatomical advantages. The posterior approach offers direct access to the posterior humeral axis but poses challenges related to the radial nerve, whose protection is critical [8], [9], [10], [11], [12], [13]. Despite the posterior approach's potential for MIPO, there is limited evidence regarding its safety, efficacy, and functional outcomes. This study aims to describe the posterior MIPO technique in detail, highlight its advantages over traditional methods, and evaluate its efficacy and safety through the functional outcomes of clinical cases. Our hypothesis is that posterior MIPO is a safe and effective alternative for distal humeral shaft fractures, associated with low complication rates and favorable functional results.

### Radial nerve anatomy and its relevance in posterior MIPO

**Fig. 1:**
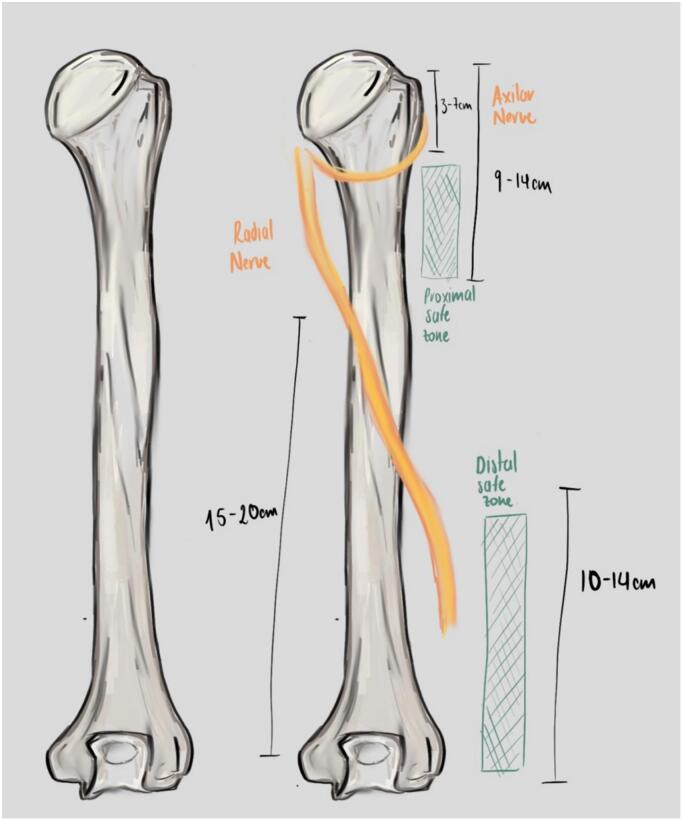
Safe zones of the posterior humerus. Proximal safe zone: 3–7 cm and 9–14 cm distal to the acromion. Distal safe zone: 10–14 cm proximal to the lateral epicondyle. *Illustration drawn by Alejandro Barros Castro, MD.*

Radial nerve protection is fundamental when performing the posterior approach to the humerus. The radial nerve, originating from the posterior cord of the brachial plexus, traverses obliquely across the posterior surface of the humerus within the spiral groove, accompanied by the deep brachial vessels [13], [14]. Anatomical studies have precisely identified its trajectory: the nerve maintains direct contact with the humerus for approximately 52.16 mm, which corresponds to 17–20% of the humeral length [14], [15], [17]. Accurate understanding and identification of “safe zones” (Fig. 1) are essential to minimize the risk of nerve injury during submuscular plate placement [13], [16], [17], [18]: Proximal Safe Zone: 3–7 cm and 9–14 cm distal to the acromion; Distal Safe Zone: 10–14 cm proximal to the lateral epicondyle.

### Indications and contraindications for posterior MIPO

Indications for posterior MIPO include multifragmentary distal humeral shaft fractures and diaphyseal-metaphyseal fractures (AO 1.2.A-c; AO 1.2.B-c; AO 1.2.C-k). This technique focuses on achieving indirect fracture reduction to promote secondary bone healing while minimizing disruption of the fracture site [8], [15]. Contraindications include simple proximal and mid-diaphyseal fracture patterns (AO 1.2.A-a and b) and fractures with neurovascular compromise [8], [15].

## Materials and methods

This retrospective case series evaluated four patients with distal third humeral shaft fractures treated with posterior minimally invasive plate osteosynthesis (MIPO). All procedures were performed by the same experienced surgical team over a 12-month period to ensure consistency in the surgical technique.

### Inclusion criteria

Patients with distal humeral shaft fractures classified as AO/OTA 1.2.A-c and 1.2.B-c (modified to describe distal third shaft fractures) [8], [15], aged between 18 and 70 years, with no prior radial nerve injury and a minimum follow-up of 12 months.

### Exclusion criteria

Patients with midshaft or proximal humeral fractures (AO/OTA 1.2.A-a-b and 1.2.B-a-b) [8], [15] or those with prior radial nerve injuries before the intervention were excluded.

### Surgical technique

**Fig. 2:**
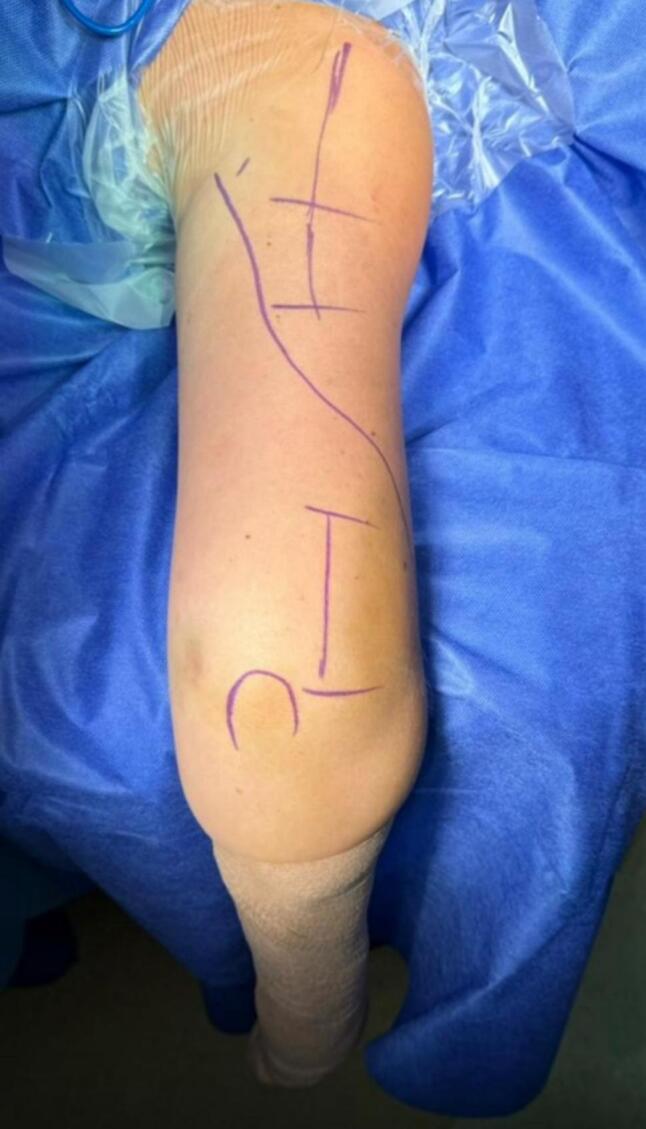
Prone positioning of the patient with the elbow flexed at 90° and the forearm hanging freely.

Patients were positioned prone on a radiolucent table. The arm was supported with folded sheets, maintaining the elbow flexed at 90° and the forearm hanging freely to facilitate indirect fracture reduction (Fig. 2). A fluoroscope was positioned at the head of the table to obtain anteroposterior and lateral images and confirm alignment before the procedure. Two longitudinal incisions of approximately 6 cm were made:

### Proximal incision

**Fig. 3:**
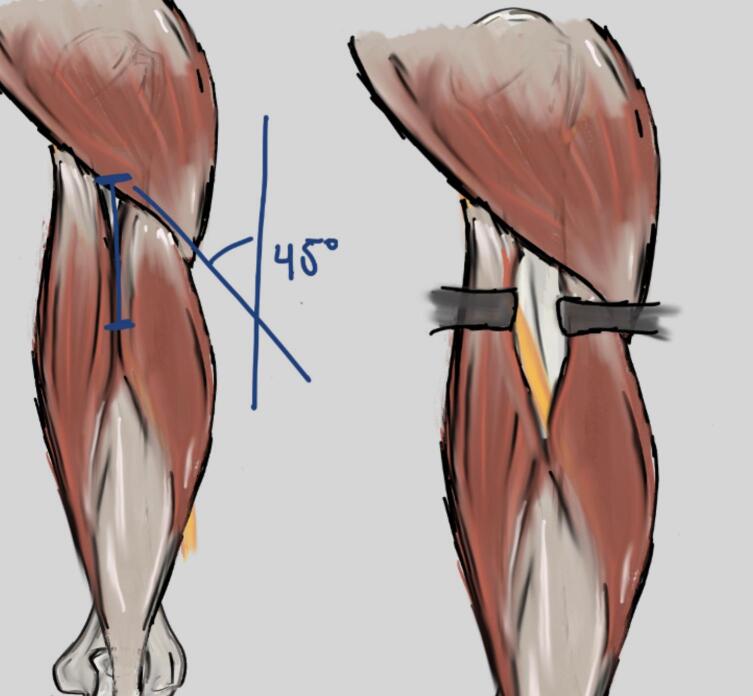
Proximal approach for the posterior MIPO technique. *Illustration drawn by Alejandro Barros Castro, MD.*

Positioned at the inferior border of the deltoid muscle belly at a 45° angle to the lateral head of the triceps. Blunt dissection between the long and lateral heads of the triceps exposed the radial nerve within the spiral groove, which was protected throughout the procedure with a vascular loop [13], [16], [17] (Fig. 3).

### Distal incision

Made 6 cm proximal to the olecranon tip. Subcutaneous dissection allowed access to the anconeus muscle, and a submuscular elevator was advanced from the distal incision to the proximal incision along the lateral column of the humerus [8], [18].

An appropriate 4.5 LCP plate (anatomical or straight) was selected and passed submuscularly from the distal to the proximal incision, crossing the fracture site and running beneath the radial nerve. Indirect reduction techniques were used to restore the fracture's length, alignment, and rotation. The plate was provisionally secured with Kirschner wires (K-wires), and its position was verified using fluoroscopy. Definitive fixation was achieved with 6 to 8 screws above and below the fracture site, using locking or cortical screws as required. In cases where the fracture gap exceeded 10 mm, a cerclage wire was applied to prevent varus or valgus deformities greater than 5° and reduce strain, following the nonunion theory proposed by Elliott et al. [19]. This approach ensured fracture consolidation within 4 months, with no cases of nonunion or pseudoarthrosis.

### Postoperative management

An elastic compression bandage and a sling were applied during the initial postoperative days to control swelling. Sutures were removed at 15 days, and no infectious complications were reported. Patients underwent clinical and radiographic follow-ups at 1 month, 6 months, and 12 months post-surgery. Shoulder and elbow range of motion was assessed, and functional outcomes were measured using validated clinical scales [8], [15], [19].

## Results

Four patients with distal third humeral shaft fractures were analyzed, classified according to a modification of the AO/OTA system. Mechanisms of injury included three cases of low-energy falls and one case related to arm wrestling. Surgical duration ranged from 1 h to 1 h 55 min, depending on the complexity of the fracture and the implant used, which included 14- to 16-hole LCP anatomical or straight plates. Only one minor complication was reported: transient paresthesia in one patient, which completely resolved within 4 weeks with no sequelae (Table 1).

**Table 1:** Patient Characteristics.

#	Laterality	Age	Sex	AO Classification	Mechanism of Injury	Surgical Time	Associated Injuries	Type of LCP Plate 4.5	Complications	Comorbidities
1	Right	32	F	1.2.B.2-c*	Fall from standing	1h15min	No	Anatomical 14-hole	Paresthesia⁎⁎	No
2	Right	39	M	1.2.A.1-c*	Arm wrestling	1h55min	No	Straight 14-hole + cerclage	No	No
3	Left	45	F	1.2.B.2-c*	Fall from standing	1h00min	No	Straight 14-hole + cerclage	No	No
4	Right	69	F	1.2.A.1-c*	Fall from standing	1h30min	No	Anatomical 14-hole	No	Rheumatoid Arthritis

AO classification modified to divide humeral shaft fractures into 3 regions: a = proximal, b = midshaft, c = distal.

⁎⁎ Resolved within 4 weeks.

### Functional outcomes

Progressive improvement in shoulder and elbow range of motion (ROM) was observed throughout follow-up evaluations at 1 month, 6 months, and 12 months postoperatively. Shoulder elevation reached 170°-180°, external rotation improved to 70°-75°, and internal rotation achieved functional levels between T4 and T5. Elbow ROM demonstrated significant progress, with flexion of 140°-145° and full extension (0°) at final follow-up. One patient, with a pre-existing comorbidity, did not regain normal ROM but still achieved functional contralateral levels similar to the other cases (Table 2, Table 3).

**Table 2:** Shoulder Range of Motion (ROM) During Follow-up.

#	Shoulder Range of Motion (ROM) 1 month *Injured/Contralateral*			Shoulder Range of Motion (ROM) 6 months *Injured/Contralateral*			Shoulder Range of Motion (ROM) 1 year *Injured/Contralateral*		
	*E*	*RE*	*RI*	*E*	*RE*	*RI*	*E*	*RE*	*RI*
1	140/180	55/75	T8/T4	160/180	60/75	T6/T4	175/180	75/75	T4/T4
2	130/175	45/80	T10/T4	170/175	55/80	T7/T4	180/175	75/80	T5/T4
3	120/180	50/75	T7/T5	160/175	60/75	T4/T5	175/175	75/75	T4/T5
4⁎	100/120	45/50	L4/L1	120/120	50/50	L3/L1	120/120	50/50	L1/L1

E: Elevation; ER: External Rotation; IR: Internal Rotation.

⁎ Patient with a history of Rheumatoid Arthritis.

**Table 3:** Elbow Range of Motion (ROM) During Follow-up.

#	Elbow ROM 1 Month *Injured/Contralateral*		Elbow ROM 6 Months 1 *Injured/Contralateral*		Elbow ROM 1 year *Injured/Contralateral*	
	*F*	*E*	*F*	*E*	*F*	*E*
1	60/140	20/0	110/140	10/0	140/140	0/0
2	80/145	30/0	120/145	10/0	145/145	0/0
3	100/145	15/0	110/145	5/0	145/145	0/0
4⁎	60/145	50/30	100/145	40/30	145/145	30/30

F: Flexion; E: Extension.

⁎ Patient with a history of Rheumatoid Arthritis.

### Functional scales

Highly favorable outcomes were reflected in validated clinical scales. VAS scores for pain were low, ranging from 0 to 2. DASH scores assessing disability ranged between 0 and 5, indicating minimal functional limitations. Additional functional assessments, such as the Constant Score and the MEPI (Mayo Elbow Performance Index), achieved values between 90 and 100 points, highlighting excellent functional recovery in both the shoulder and elbow (Table 4).

**Table 4:** Functional Outcomes.

	*VAS*	*DASH*	*Constant* *score*	*MEPI*	*Angulation (°) Varus/Valgus*	Follow-up Duration
1	1	5	95	90	5° varo	12 months
2	0	0	100	100	5° varo	18 months
3	0	0	100	100	0°	20 months
4*	2	5	95	90	0°	36 months

VAS: Visual Analog Scale; DASH: Disabilities of the Arm, Shoulder, and Hand; MEPI: Mayo Elbow Performance Index. *Patient with a history of Rheumatoid Arthritis.

### Radiographic findings

Radiographic evaluations demonstrated proper alignment at final follow-up, with residual varus angulation between 0° and 5°, which had no clinical impact on patient functionality.

**Fig. 4:**
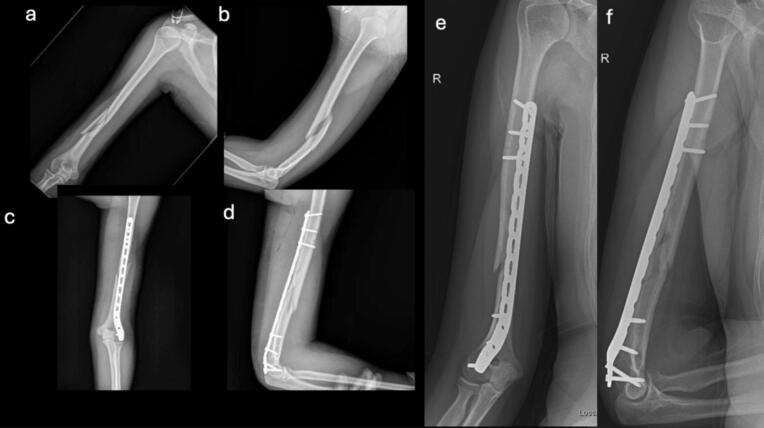
Case 1 – Female patient, 35 years old, mechanism of injury: fall from standing height. a-b) Preoperative AP and lateral radiographs showing AO 1.2.B.2-c fracture; c-d) Immediate postoperative AP and lateral radiographs; e-f) AP and lateral radiographs at 1-month follow-up.

**Fig. 5:**
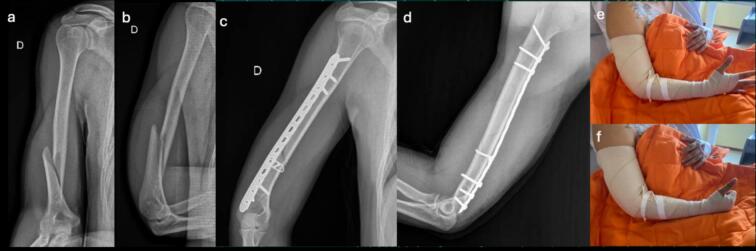
Case 2 – Male patient, 31 years old, mechanism of injury: arm wrestling. a-b) Preoperative AP and lateral radiographs showing AO 1.2.A.1-c fracture; c-d) Immediate postoperative AP and lateral radiographs; e-f) Immediate postoperative evaluation of radial nerve motor function.

**Fig. 6:**
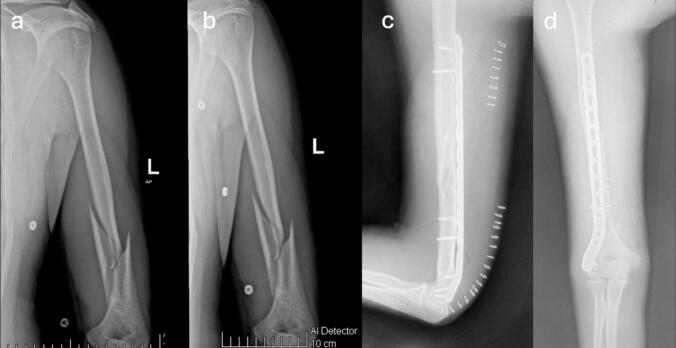
Case 3 – Female patient, 45 years old, mechanism of injury: fall from standing height. a-b) Preoperative AP and lateral radiographs showing AO 1.2.B.2-c fracture; c-d) Immediate postoperative AP and lateral radiographs; e-f) AP and lateral radiographs at 1-month follow-up.

**Fig. 7:**
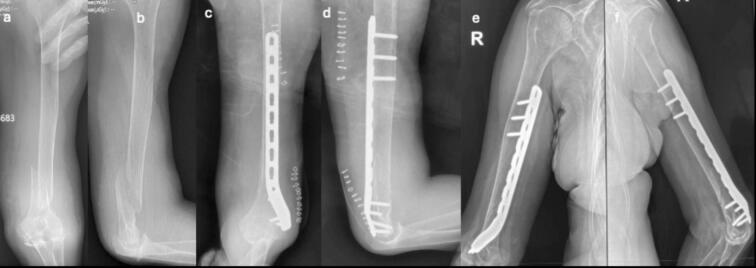
Case 4 – Female patient, 69 years old, mechanism of injury: fall from standing height. a-b) Preoperative AP and lateral radiographs showing AO 1.2.A.1-c fracture; c-d) Immediate postoperative AP and lateral radiographs; e-f) AP and lateral radiographs at 1-month follow-up.

**Fig. 8:**
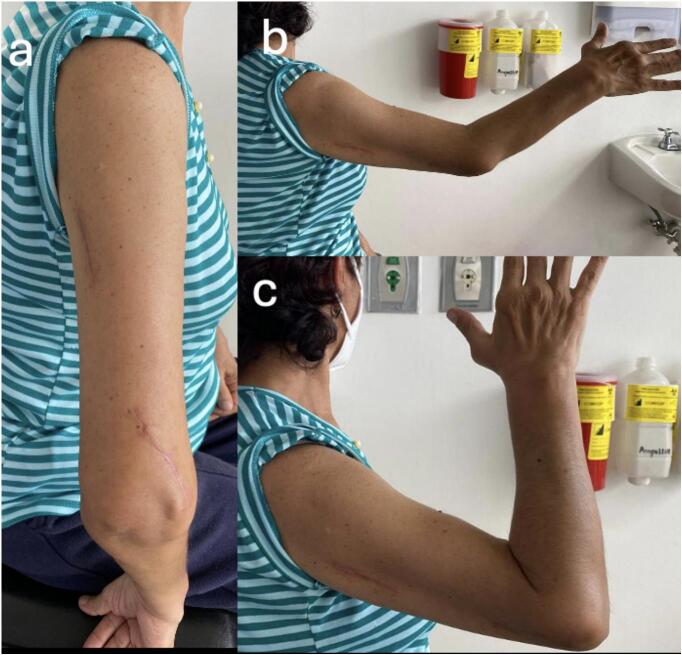
Case 4 – Female patient, 69 years old. a) Surgical wounds at 1-month follow-up; b) Elbow extension of 30° at 1-year follow-up; c) Elbow flexion of 145° at 1-year follow-up.

These findings demonstrate positive evolution in terms of joint mobility, bone alignment, and functionality, with a low incidence of complications and good implant tolerance. Collectively, the results support the safety and efficacy of the posterior MIPO technique as a viable option for treating distal third humeral shaft fractures (Fig. 4, Fig. 5, Fig. 6, Fig. 7, Fig. 8).

## Discussion

Posterior Minimally Invasive Plate Osteosynthesis (MIPO) has emerged as a promising technique for managing distal third humeral shaft fractures by combining minimal soft tissue disruption with a reduced rate of complications compared to traditional methods. However, its implementation presents unique challenges, particularly in radial nerve protection and optimization of fracture reduction, both critical for surgical success.

In our case series, the results obtained align with the available literature, reinforcing the viability and safety of posterior MIPO when performed with surgical precision. Gallucci et al. [9], [10] pioneered this approach, reporting excellent functional outcomes. In their initial study of 15 patients with midshaft humeral fractures, complete consolidation was achieved in all cases. Although one patient developed transient radial nerve palsy, it fully resolved within 6 weeks, confirming the low neurological risk when the radial nerve is adequately protected. Their second study, which focused on distal third humeral shaft fractures in 21 patients, showed similarly encouraging results, with an average Constant Score of 84 and only one additional case of temporary neuropraxia. These findings highlight the consistency of the technique in delivering good functional outcomes with minimal neurological complications.

Similarly, Ballam et al. [11] evaluated 37 patients, observing progressive functional recovery using the Quick DASH scale, which improved from 64 to 34 points within the first 6 months and achieved full functionality at 1 year. While two cases of delayed union required shockwave therapy, resolution was satisfactory. Furthermore, only two patients developed transient radial neuropraxia, further confirming the low risk of neurological complications when meticulous technique is applied.

In a more recent comparative study, Boretto et al. [12] analyzed 67 patients treated with posterior MIPO, dividing them into two groups based on implant type: straight LCP plates versus anatomical plates. Although both groups achieved full bone union, anatomical plates provided a significant advantage by reducing implant intolerance due to their improved conformity with humeral geometry. This observation underscores the importance of implant selection in optimizing postoperative outcomes.

When analyzing cumulative data from the reviewed studies (Table 5) [9], [10], [11], [12], which included a total of 140 patients, the incidence of radial nerve neuropraxia was 4.29% (6 cases in total), all of which resolved spontaneously within 6 to 8 weeks. These findings reflect that, although the risk of neurological injury exists, it is low and transient when strict protocols for radial nerve identification and protection are followed during surgery.

**Table 5:** Summary of Published Studies Using Posterior MIPO and Radial Nerve Complications.

Study	Number of Patients	Radial Nerve Neuropraxia⁎
Gallucci et.al [10]	15	1
Gallucci et.al [9]	21	1
Ballam [11]	37	2
Boretto et al. [12]	67	2
***Total***	***140***	***6***

⁎ Transient, resolved within 6 weeks; NR: Radial Nerve.

Currently, the use of complementary tools, such as preoperative ultrasound [18] and magnetic resonance imaging (MRI) [20], represents an innovative strategy to precisely identify the radial nerve trajectory, enabling safer and more effective surgical planning. These technologies may further reduce the risk of iatrogenic complications, particularly in complex cases where anatomical variations of the nerve are difficult to predict.

The posterior MIPO approach offers several significant advantages over traditional techniques. It minimizes soft tissue disruption by reducing dissection and surgical manipulation, decreases the risk of nonunion and pseudoarthrosis by preserving periosteal blood supply, and provides direct anatomical access to the posterior humeral axis, facilitating submuscular implant placement. Importantly, posterior MIPO is associated with a reduced incidence of radial nerve palsy compared to open techniques. These advantages establish posterior MIPO as a safe and efficient alternative, particularly for distal third humeral shaft fractures, where traditional approaches may face anatomical and functional limitations.

## Conclusions

The findings of this study, along with existing evidence, demonstrate that the posterior MIPO technique is a viable and safe surgical option for managing distal third humeral shaft fractures. The low incidence of complications, satisfactory functional recovery, and effective bone consolidation support its implementation in clinical practice. However, the success of this technique relies on the accurate identification and protection of the radial nerve, as well as the use of complementary tools such as ultrasound or MRI in complex cases. Future studies with larger sample sizes and long-term follow-up will be essential to validate these results and establish posterior MIPO as a standard of care for distal humeral fractures.

## CRediT authorship contribution statement

**Edgar Barros Prieto:** Conceptualization. **Carlos Ballesteros Ponce:** Conceptualization, Investigation. **Carlos Eduardo Noboa Freile:** Investigation. **Carlos Peñaherrera Carrillo:** Investigation. **Francisco Endara:** Methodology, Resources. **Paul Santiago Vaca:** Writing – original draft, Writing – review & editing. **Diego Michilena:** Visualization, Writing – original draft, Writing – review & editing. **Alejandro Xavier Barros Castro:** Conceptualization, Data curation, Formal analysis, Investigation, Methodology, Project administration, Writing – original draft, Writing – review & editing.

## Informed consent

All images and photographs included in this manuscript were obtained with the written and signed informed consent of the patients.

## Ethical approval

This study was conducted in compliance with the ethical standards outlined in the Declaration of Helsinki. The research protocol was approved by the Institutional Review Board (IRB) of the participating institution.

## Declaration of Generative AI and AI-assisted technologies in the writing process

No AI tools were used in the writing, analysis, or preparation of this manuscript.

## Funding disclosure

The authors declare that no funding was received for this research.

## Declaration of competing interest

The authors declare no conflicts of interest related to this study.
